# A Case of Catarrhal Pneumonia in Childhood

**Published:** 1894-05

**Authors:** Albert S. Miller

**Affiliations:** Athens, Texas


### A CASE OF CATARRHAL PNEUMONIA IN CHILDHOOD.

BY ALBERT S. MILLER, N.G., M.D., ATHENS, TEXAS.

I desire to call attention to this case for three reasons: 1st. It was the second attack of pneumonia the child had had this year. 2d. The intensity of the symptoms in both cases; and 3d, the use of strychnine as part of the treatment.

On the evening of the 14th of March, at 7 o'clock, I was called to see this case. Child of Mr. and Mrs. P. W., male, white, aged 12 or 14 months, of robust constitution and pure family history. I found the little sufferer with a temperature of 103½, pulse quick and full, respiration not abnormally fast for the temperature. The skin was dry and hot, bowels constipated, lips dry, and a look of anxiety about the face. Patient had had more or less fever during the day, which possibly it contracted by slight exposure the evening before. There was a great deal of cold on the lungs, but physical examination gave no signs of pneumonia. In fact, I could not have expected chest signs so early in this form of the disease.

Knowing that the little patient had just recovered from a severe attack of pneumonia contracted in the first part of January, I realized that his lungs were not in a perfect condition and would be more susceptible than otherwise to deep inflamation of their structure. So I prescribed the following with a view of arresting the fever and any tendency to pneumonia if such disposition was present:

NUMBER ONE

℞ Hydrarg chlor. mit............................................gr. iii.
Pulv. opii. et ipecac ............................................
Pot. nitrat..................................................ā ā. gr. i.
M.—Triturate for one powder only.
Sig.—Given at once.

Also I prescribed a drachm of febriline to be given at 8 o'clock, and half this quantity to be given at 11 o'clock same evening, and to be followed next morning at 6 A. M. by another drachm dose.

March 15th, 9 A. M., temperature 101⅘, pulse rapid but regular, one cheek slighly flushed, an occasional repressed cough and pain on being moved. Physical signs were now slightly manifested. There was slight bronchial breathing and fine moist râles about the base of both lungs and especially that of the left. Patient had rested moderately well during the night and had two or three good actions from the bowels that morning. I had the chest covered with a flannel well oiled with lard and turpentine, and continued half drachm doses febriline four hours apart until I would see my patient again at 6 P. M. At this time the temperature was 104, respirations 52, pulse rapid, cheeks flushed, a labored cry when disturbed and physical signs of the morning highly magnified. The left lung showed a more general effusion throughout its lower part than the corresponding section of the right. I now gave one and one-half grains phenacetine to reduce nervous tension and prescribed the following:

NUMBER TWO.

℞ Spts. ammonii ar.........................................ʒ ii.

Spts. athuis nitrasi.........................................ʒ i.

Tr. aconitæ rad.........................................gr. viii.

Pot. acetat.........................................gr. iii.

Aq. puræ q. s.........................................℥ iii.

M. Sig. Teaspoonful 2 hours apart.

I reduced the quantity of calomel in No. 1 one-half and made three powders which I directed to be given three hours apart during the night. Continued half drachm doses febriline four hours apart and requested nurse in case patient was resting quietly at 12 o'clock when all three powders had been taken to discontinue medicines until 6 A. M. I also directed nourishment, principally buttermilk to be given every two or three hours.

March 16th, 9 A. M., I found temperature 102½, pulse 140, respirations 46, and flush in cheeks not so perceptible. Patient had rested very well during the night and bowels acted well in the morning. I did not examine chest for I had it covered with oiled flannel and deemed it unnecessary, as I knew already what I had to deal with. I left a powder of phenacetine 1½ grains to be given in case fever ran high enough to create restlessness and continued No. 2 and half drachm doses of febriline four hours apart. I was not to return until next morning.

March 17th, 9 A. M., temperature 103⅗, pulse 160, respirations,

54, cheeks flushed, patient restless, a rattle in breathing, skin dry, lips cracked and patient very fretful on being moved. I knew I had considerable extension of effusion into new lobules but did not make physical examination to find how much was involved. I gave my patient a hot mustard bath for about ten minutes. This reduced fever to 102 and restored rest. Continued No. 2 and half drachm doses febriline six hours apart and prescribed two or three teaspoonfuls of a mild eggnog every two hours. I again saw my patient at 5 P. M., temperature 104½, and other symptoms a little more intense than they were that morning. I gave an enema of hot soap suds and after bowels acted off and patient was rested from the worry, I also gave one and one-half grains phenacetine and continued morning treatment during the night, recommending patient's body to be sponged well next morning with warm water.

March 18th, 9 A. M., temperature 103½, pulse 158, respirations 56, and irregular, with distinct rattling, cheeks slightly flushed, bowels distended, face pale, skin dry, and patient very weak; had a restless night and took but little nourishment. I gave No. 1 reduced one-half in quantity at once and a hot mustard bath for ten minutes; doubled ammonia in No. 2 and continued it and febriline as before directed. I left one and a half grains phenacctine to be given at 12 A. M. I saw my patient again at half past seven o'clock that evening. Temperature 105, pulse 178, respiration, 72, irregular and labored. There was considerable effusion into the large bronchi and a loud rattling noise made in breathing. I gave one and one-half grains phenacetine and after a bit began one-hundredth grain nitrate strychnine every four hours. The respirations soon fell to fifty-eight and were much stronger. I applied hot, wet cloths to the chest and bowels for half or an hour at a time several times during the night and gave an enema of soap suds at 10 : 30. Continued febriline and No. 2 as last prescribed and gave all the eggnog patient could bear. He spent a very restless night, but all the symptoms gradually toned down as the night escaped.

March 19th, 9 A. M., temperature 101, pulse rather weak, respirations 58 and strong, secretions acting nicely and patient resting quietly. Patient was better in every respect and resolution was set in. Had body sponged with warm water, and made no change in treatment. Afternoon at 4 o'clock, temperature 100, respirations 54 and strong, treatment unaltered.

March 20th, 9 A. M., temperarure 98½, respirations 42. All other symptoms rapidly improving; left off strychnine and continued febriline and No. 2 four hours apart only during the day. Dismissed, my patient doing well, and he made a rapid recovery.

*Remarks.*—This child had a very similar attack of catarrhal pneumonia two months preceding this attack, but had regained about all the weight and strength he lost from first attack when second attack set up. The line of treatment in first attack was about the same as that laid down for last, except tinct. nux vomica was given instead of strychnine to tone respiratory action.

In regard to strychnine, I desire to say I use it in most all my cases of pneumonia on the home stretch, and believe I get good results from its use, but like other stimulants, I think it should not be given too early. I feel safe in saying it is a great respiratory stimulant; not, however, one that would increase the respiratory efforts, but that which would slow, regulate and make them deeper. In pneumonia, especially the catarrhal form of the disease, the physical forces are usually exhausted oftener by overdone respiratory effort than by tax on the heart's action. The respirations toward the crisis of pneumonia becomes rapid, irregular, shallow and labored; these symptoms are due to two conditions, limited breathing area and a toxic state of the respiratory centers wherein they lose control of their power, and as we may say, turn loose the respiratory functions, thus giving rise to the above recorded symptoms. There are conditions in which I think strychnine serves an efficient purpose, at least I give it in addition to good eggnog, and if my patient fails to pull through, I feel that my practice has been scientific at least.

